# Structural investigation into physiological DNA phosphorothioate modification

**DOI:** 10.1038/srep25737

**Published:** 2016-05-12

**Authors:** Wenxian Lan, Zhongpei Hu, Jie Shen, Chunxi Wang, Feng Jiang, Huili Liu, Dewu Long, Maili Liu, Chunyang Cao

**Affiliations:** 1 State Key Laboratory of Bio-organic and Natural Product Chemistry, Shanghai Institute of Organic Chemistry, Chinese Academy of Sciences, 345 Lingling Road, Shanghai, 200032, China; 2 Tianjin Institute of Industrial Biotechnology, Chinese Academy of Sciences, 32 Xiqidao, Tianjin Airport Economic Area, Tianjin, 300308, China; 3 Shanghai Institute of Applied Physics, Chinese Academy of Sciences, 2019 Jialuo Road, Shanghai, 201800, China; 4 State key Laboratory of Magnetic Resonance and Atomic and Molecular Physics, Wuhan Institute of Physics and Mathematics of Chinese Academy of Sciences, West No.30 Xiao Hong Shan, Wuhan 430071, China

## Abstract

DNA phosphorothioate (PT) modification, with sulfur replacing a nonbridging phosphate oxygen in a sequence and stereo specific manner, is a novel physiological variation in bacteria. But what effects on DNA properties PT modification has is still unclear. To address this, we prepared three double-stranded (ds) DNA decamers, d(CG^PX^GCCGCCGA) with its complementary strand d(TCGGCG^PX^GCCG) (where X = O or S, *i.e.*, PT-free dsDNA, [*S*_p_, *S*_p_]-PT dsDNA or [*R*_p_, *R*_p_]-PT dsDNA) located in gene of *Streptomyces lividans*. Their melting temperature (*T*_m_) measurement indicates that [*R*_p_, *R*_p_]-PT dsDNA is most unstable. Their electron transfer potential detection presents an order of anti-oxidation properties: *S*_p_-PT DNA > *R*_p_-PT DNA > PT-free DNA. Their NMR structures demonstrate that PT modification doesn’t change their B-form conformation. The sulfur in [*R*_p_, *R*_p_]-PT dsDNA locates in the major groove, with steric effects on protons in the sugar close to modification sites, resulting in its unstability, and facilitating its selectively interactions with ScoMcrA. We thought that PT modification was dialectical to the bacteria. It protects the hosting bacteria by working as antioxidant against H_2_O_2_, and acts as a marker, directing restriction enzyme observed in other hosts, like ScoMcrA, to correctly cleave the PT modified DNA, so that bacteria cannot spread and survive.

DNA phosphorothioate (PT) modification is a novel physiological variation in a specific sequence and stereo (*i.e*., *R*_p_-PT) manner (Fig. 1A–C), in which sulfur is incorporated into DNA backbone^1^. The DNA PT modification is controlled by the five-gene *dnd* cluster (*dndA-E*) products^2^,^3^,^4^,^5^. The *dndA* and *dndC-E* genes are necessary for PT modification, while *dndB* inactivation increases phosphorothioation and alters sequence preference^6^. DndA is a cysteine desulfurase with a catalytic cysteine located on its rigid β strand, and assembles DndC as a 4Fe-4S cluster protein^7^. DndB is a homolog to a group of transcriptional regulators^5^. DndC has ATP pyrophosphatase activity, as well as 3′-phosphoadenosine-5′-phosphosulfate (PAPS) reductase activity^6^,^8^. DndD has ATPase activity possibly related to DNA structure alteration or nicking during PT incorporation^9^. DndE acts as a nicking dsDNA binding protein^10^. During PT modification, DndA, DndC, DndD and DndE form a complex to perform oxygen-sulfur switch, while DndB displays little or weak binding affinity to the complex^11^.Within *E. coli* strain, the Dnd operon for DNA PT modification system is located in diverse genomic islands^12^, facilitating the horizontal gene transfer.

The DNA PT modification results in DNA degradation (Dnd) phenotype (*i.e.*, the *R*_p_-PT DNA is degraded by oxidative cleavage during electrophoresis in tris-acetate buffer)^3^. It is widespread and quantized in bacterial genomes. Working as a part of a restriction modification system^2^,^3^,^9^, it can be specifically cleaved *in vitro* by type IV modification-dependent restriction endonuclease^5^,^13^. The *R*_p_-PT DNA functions as an antioxidant, which protects the hosting bacteria against peroxide^14^. However, it still remains unclear that how PT modification affects DNA structure and function.

To address this, here, we synthesized and purified three double-stranded (ds) DNA decamer, *i.e.*, the strand d(C_1_G_2_^PX^G_3_C_4_C_5_G_6_C_7_C_8_G_9_A_10_) with its complementary strand d(T_11_C_12_G_13_G_14_C_15_G_16_^PX^G_17_C_18_C_19_G_20_) (where X = O or S, thus, hereinafter, the dsDNA were named as PT-free dsDNA, [*S*_p_, *S*_p_]-PT dsDNA, or [*R*_p_, *R*_p_]-PT dsDNA, respectively) obtained from *Streptomyces lividans*. To probe relative stability and redox potential of PT modified dsDNA, we measured their melting temperatures (*T*_m_) by differential scanning calorimetry (DSC) assay, and electron transfer potential by gold electrodes. To investigate the possible conformational changes of duplexes by PT modification, we determined NMR solution structures of PT-free dsDNA, [*S*_p_, *S*_p_]-PT dsDNA, and [*R*_p_, *R*_p_]-PT dsDNA through conventional two-dimensional (2D) NMR techniques.

## Results and Discussion

### [R_p_, R_p_]-PT modification destabilizes B-form dsDNA

**Figure 1:**
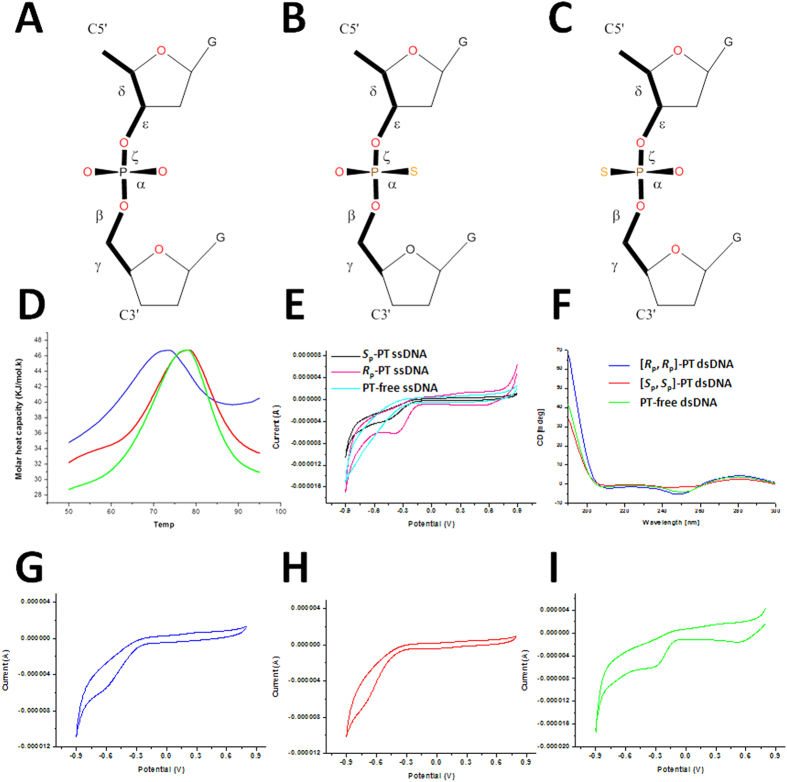
The properties of PT-dsDNA. (**A**–**C**) The chemical structure of PT-free, *R*_p_-PT and *S*_p_ -PT modification in DNA, respectively. The torsion angles were indicated. (**D**) The melting temperature of PT-free dsDNA (green), [*S*_p_, *S*_p_]-PT dsDNA (red), and [*R*_p_, *R*_p_]-PT dsDNA (blue) measured by DSC assay. (**E**) The electron transfer potentials of ssDNA measured by cyclic voltammetry assay of *S*_p_-PT ssDNA (black), *R*_p_-PT ssDNA (pink) and PT-free ssDNA (cyan). (**F**) The circular dichroism (CD) spectra of PT-free dsDNA (green), [*S*_p_, *S*_p_]-PT dsDNA (red), [*R*_p_, *R*_p_]-PT dsDNA (blue), respectively; (**G**–**I**) The electron transfer potentials of dsDNA containing buffer control, PT-free dsDNA (green), [*S*_p_, *S*_p_]-PT dsDNA (red), [*R*_p_, *R*_p_]-PT dsDNA (blue).

PT modified DNA isolation and P-S bond stereochemistry characterization were described in supplemental material and methods. To probe the relative stability of dsDNA with or without PT modifications in the sequences, we measured their melting temperature (*T*_m_) by differential scanning calorimetry (DSC) assay (Fig. 1D). In this assay, the area under the calorimetrically measured excess heat capacity (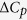) versus temperature profiles is proportional to the total endothermic heat (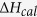) needed to disrupt these duplexes into singe strands. Compare to PT-free dsDNA (*T*_m_ = 76.55 ± 0.78 °C), the thermal stability of [*R*_p_, *R*_p_]-PT dsDNA was partially reduced (*T*_m_ = 71.99 ± 0.78 °C, decreased by about 4.5 degree), consistent with the recently reported theoretical prediction^15^. On the contrary, the stability of [*S*_p_, *S*_p_]-PT duplex (*T*_m_ = 77.53 ± 0.58 °C) is slightly better than that of the PT-free dsDNA (*T*_m_ is increased only by about 1 degree). Except for the PT modification sites, the sequences of the remaining parts of these duplexes are identical. We thus conclude that the PT modification leads to the differences in the melting temperature.

Our [*S*_p_, *S*_p_]-PT duplex is more stable than the biological [*R*_p_, *R*_p_]-PT dsDNA, identical with the previously observed results of synthesized PT-modified dsDNA (hereinafter, to clearly differentiate it from our PT-modified dsDNA, we named it as PT DNA-DNA) with a sequence of d(GC^PS^T^PS^ACG)^16^. In this PT DNA-DNA, the two PT modification sites are ocurred only on one strand d(GC^PS^T^PS^ACG). However, this [*S*_p_, *S*_p_]-PT modified DNA-DNA is less stable than its unmodified DNA-DNA. That is to say, the stability of this PT DNA-DNA is: PT-free DNA-DNA > [*S*_p_, *S*_p_]-PT DNA-DNA > [*R*_p_, *R*_p_]-PT DNA-DNA, much different from our PT-modified dsDNA. When this complementary unmodified DNA strand was switched into a RNA strand (*i.e.*, PT DNA-RNA), the [*R*_p_, *R*_p_]-PT modified DNA-DNA is more stable than the [*S*_p_, *S*_p_]-PT modified DNA-RNA. Thus, sometimes, it seems that the stability of PT modified DNA is related to not only electrostatic or steric reasons, but also the sequential composition^17^.

### R_p_-PT DNA demonstrates electron transfer potential weaker than S_p_-PT DNA

To probe why the PT-modified DNA can act as an antioxidant in bacteria^14^, the electron transfer (ET) potentials were measured to investigate the redox potential of PT-free duplex, [*S*_p_, *S*_p_]-PT duplex and [*R*_p_, *R*_p_]-PT duplex by cyclic voltammetry assay (Fig. 1). Both ET potentials of [*R*_p_, *R*_p_]-PT dsDNA (−0.62 V) and [*S*_p_, *S*_p_]-PT dsDNA (−0.72 V) are better than that of PT-free dsDNA (−0.28 V). Moreover, the *R*_p_-PT modified ssDNA has ET potential (−0.37 V) slightly weaker than the *S*_p_-PT modified ssDNA (−0.43 V) (Fig. 1E and supplemental Table S1), while the ET potential of PT-free ssDNA is too weak to be detected. These results can be explained by the subtle changes produced by the PT modification on DNA backbone. Compared to normal prochiral phosphate (where the negative charge is delocalized across the two phosphoryl oxygen atoms), the phosphorothioate group has predominant resonance form (O = P-S^−^)^18^, where the negative charge is more localized in sulfur atom than normal phosphate. In a phosphorothioate, the bond lengths are different and charge is more localized than that in a prochiral phosphate. These changes present a sharp probe of the interaction with individual phosphates. Therefore, either PT-modified dsDNA or ssDNA has better redox potential than the corresponding PT-free dsDNA or ssDNA.

### NMR spectra analysis

To probe the PT modification effects on dsDNA structure, we decided to perform NMR studies on PT-free dsDNA, [*S*_p_, *S*_p_]-PT dsDNA, and [*R*_p_, *R*_p_]-PT dsDNA. NMR spectra were analyzed using program Sparky. All nonexchangeable protons of three DNA duplexes were assigned using established techniques for right-handed dsDNA using DQF-COSY and 2D NOESY spectra^19^,^20^. The methyl group in sole thymine (T_11_ in the sequence) was first identified, thus working as a starting point of the assignment. Then, the T_11_ H6 resonance was assigned by strong NOE connectivity in NOESY spectrum. The resonances of H5/H6 of each cytosine (*i.e*., C_1_, C_4_, C_5_, C_7_, C_8_, C_12_, C_15_, C_18_ and C_19_ in the sequence) were easily recognized in both TOCSY and COSY spectra, much different from H8 of guanines. The resonances in the deoxyribose sugars were identified by their strong NOE peaks with H1′ and confirmed by the cross-peaks with the corresponding base proton (*i.e*., H6/H8 in the base with H1′ in sugar). Differences in the intensities of the cross-peaks of H1′ with H2′ and H2″ at short mixing time were used to do stereo-specifically assignments of H2′ and H2″ resonances. Spin systems in the sugar moiety of the DNA strand were identified in the DQF-COSY spectra.

For both strands, assignments of the cross-peaks connecting each base with its own sugar and the 3′-neighbor were followed in the base-H1′ or base-H2′/H2″ region, including sequential connectivities with the 3′-linked nucleotide and inter-strand contacts with the base-paired nucleotide to the 5′-neighboring residue. For example, the NOE connectivities between H6/H8 in base of residue *i* and H2′/H2″ or H1′ in sugar of residue *i* − 1 were used for sequential assignment. The exchangeable imino H3 proton in T_11_ was identified by their strong inter-strand NOE cross-peak with H2 of the base-paired A_10_. Labile protons in GC base pairs were assigned by following their connection to H5 of cytosine. Except for the terminal residues, all imino protons were identified. In this way, including all H4′ and most of the H5′ and H5″, almost all resonances of nonexchangeable and exchangeable protons were assigned. The resonances of ^31^P were assigned using cross-peaks in HECTOR spectrum between ^31^P and H4′/H5′ within the residue or H3′ in 5′-neighboring residues.

**Figure 2:**
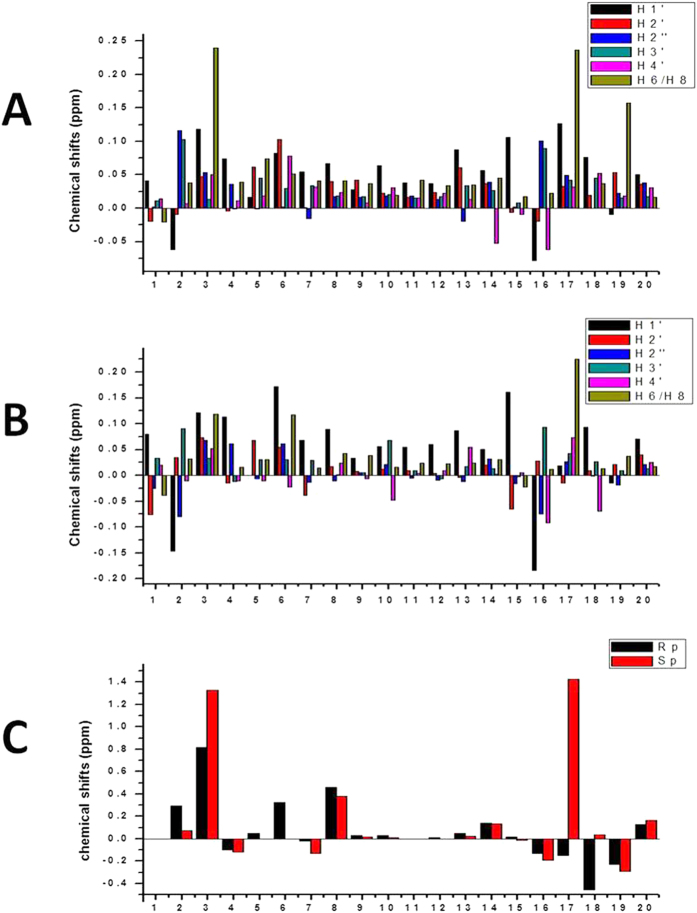
The NMR chemical shift differences (Δδ = δ_PT_ − δ_PT−free_) among PT-free dsDNA, [Rp, Rp]-PT dsDNA and [Sp, Sp]-PT dsDNA. (**A**) The chemical shift difference of protons in the bases between PT-free dsDNA and [*R*_p_, *R*_p_]-PT dsDNA, (**B**) The chemical shift difference of protons in the bases between PT-free dsDNA and [*S*_p_, *S*_p_]-PT dsDNA. (**C**) ^31^P chemical shift difference between PT-free dsDNA and [*R*_p_, *R*_p_]-PT dsDNA (black), and between PT-free dsDNA and [*S*_p_, *S*_p_]-PT dsDNA (red), respectively.

Compared to the PT-free dsDNA, both stereoisomers display almost similar chemical shifts of protons (Fig. 2). Except G_3_ and G_17_ (their H8 protons have chemical shift changes larger than 0.2 ppm), most protons have chemical shifts variation less than 0.2 ppm. Thus, the global structures of the duplexes might be identical to that of PT-free dsDNA. Large differences in chemical shifts were mostly located very close to the phosphorothioation sites in two strands: G_2_^PS^G_3_C_4_ and G_16_^PS^G_17_C_18_. Specifically, compared to phosphorus atoms of G3 and G17 (with the chemical shifts of −0.86 ppm and −0.89 ppm, respectively) of PT-free dsDNA, the phosphorus atoms of modified sites G_3_ and G_17_ of [*R*_p_, *R*_p_]-PT dsDNA display chemical shifts at −0.04 ppm and −0.19 ppm, respectively, while the phosphorus atoms of modified sites G_3_ and G_17_ of [*S*_p_, *S*_p_]-PT dsDNA demonstrate chemical shifts at 0.47 ppm and 0.54 ppm, respectively. Thus, the perturbation on the chemical shifts of phosphorus atoms in the modification sites in [*R*_p_, *R*_p_]-PT dsDNA is smaller than that in [*S*_p_, *S*_p_]-PT dsDNA. These observations may be resulted from the electrostatic effects, in which the negative charge in a normal prochiral phosphate is more delocalized than that in a phosphorothioate group.

### NMR solution structures analysis

**Figure 3:**
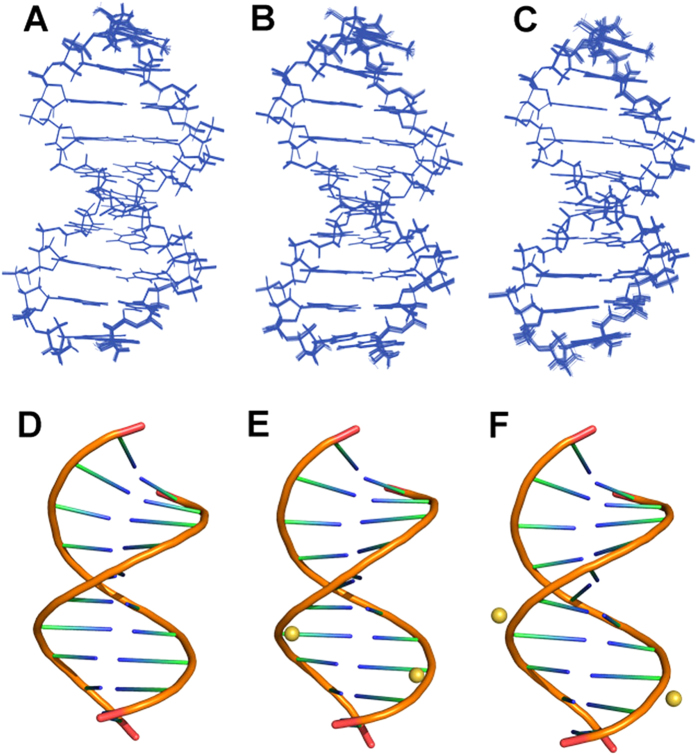
The solution structures of (**A**,**D**) PT-free dsDNA, (**B**,**E**) [*R*_p_, *R*_p_]-PT dsDNA and (**C**,**F**) [*S*_p_, *S*_p_]-PT dsDNA, displayed in their 20 structures ensembles in a line mode (**A**–**C**) and in a cartoon mode (**D**–**F**), respectively. The balls represent the sulfur atoms in the PT-modified dsDNA.

**Figure 4:**
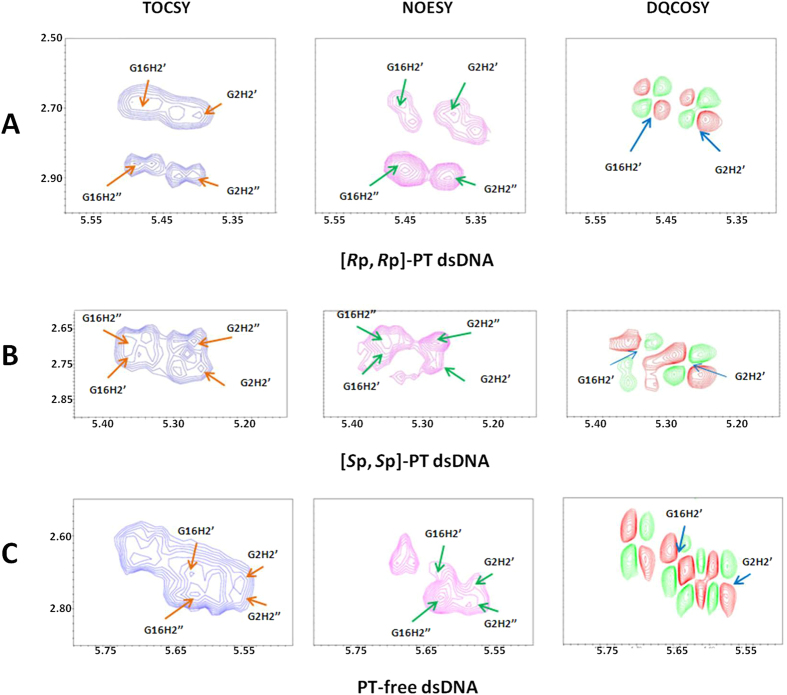
The B-helix conformation of dsDNA determined by *J*-coupling constants of ^3^*J*_H1′–H2′_ (>10 Hz) in 2D NMR ^1^H-^1^H DQF-COSY spectra (right). The assignments of the cross-peaks H1′-H2′ of each residues in the DNA sequences were performed based on the spin system identification in ^1^H-^1^H TOCSY and NOE intensity differences between the cross-peaks of H1′-H2′ and H1′-H2″ in ^1^H-^1^H NOESY spectrum. Here, we used the assignments of H1′-H2′ of residues G2 and G16 as examples. (**A**) [*R*_p_, *R*_p_]-PT dsDNA, (**B**) [*S*_p_, *S*_p_]-PT dsDNA and (**C**) PT-free dsDNA. The assignments of all H2′ and H2″ protons in sugar ring were performed based on the spin system identification through 2D NMR ^1^H-^1^H TOCSY spectra (left), and confirmed by the intensity of NOE cross peaks between H1′ and H2′ or H2″, observed in 2D NMR ^1^H-^1^H NOESY spectra (middle).

To probe how the PT modification effects on dsDNA structure, we determined NMR solution structures of PT-free dsDNA, [*S*_p_, *S*_p_]-PT dsDNA, and [*R*_p_, *R*_p_]-PT dsDNA (Fig. 3, Table 1). The structures of [*S*_p_, *S*_p_]-PT dsDNA, [*R*_p_, *R*_p_]-PT dsDNA are almost similar to that of PT-free dsDNA with RMSD values of 1.23 Å and 0.58 Å, respectively, upon aligning PT modified dsDNAs with PT-free dsDNA. This observation accords with the results from circular dichroism (CD) spectra, where all dsDNAs have conservatively positive absorption at about 220 nm and 275 nm, and negative absorption at about 245 nm, as shown in Fig. 1F. All dsDNA have a B-form duplex conformation, consistent with the measurement of *J*-coupling values (>10 Hz, indicating S-type conformer of B-form) between H1′ and H2′ protons in sugar ring (Fig. 4). In [*R*_p_, *R*_p_]-PT dsDNA, the sulfur atoms locate inside of the major grooves of dsDNA (Fig. 3E), On the contrary, in [*S*_p_, *S*_p_]-PT dsDNA (Fig. 3F), the charged phosphorothioate groups are fully exposed to the solvent, and the P-S bonds point to the outside of the major groove of the dsDNA. The location sites of sulfur atoms in the surface of [*S*_p_, *S*_p_]-PT dsDNA facilitate their interactions with Ag/AgCl electrode, resulting in the enhancement of the electron transfer, which coincides with the measurement of electron transfer potential above.

In the topology of [*R*_p_, *R*_p_]-PT dsDNA, the original oxygen atom O2P connecting phosphorus is switched by sulfur atom, while in the topology of [*S*_p_, *S*_p_]-PT dsDNA, the original oxygen atom O1P linking phosphorus is changed by sulfur atom (Fig. 1A–C). In 20 lowest-energy structures of PT-free dsDNA and [*S*_p_, *S*_p_]-PT dsDNA (Fig. 3A–C), the averaged distances between the proton H2″ in sugar ring of residue G2 or G16 and the neighboring oxygen atom O2P of residue G3 or G17 were measured as 2.47 Å and 2.39 Å, respectively. The averaged distance between the proton H2″ in sugar ring of residue G2 or G16 and the adjacent sulfur atom of residue G3 or G17 in [*R*_p_, *R*_p_]-PT dsDNA was 2.60 Å. These observations indicate that the change from the attractive C-H•••O contact to the repulsive C-H•••S contact in [*R*_p_, *R*_p_]-PT dsDNA is unfavorable in B-helix, due to short-range H•••S interaction. The less flexibility of torsion angles α and ζ further enhances this disadvantageous interaction, resulting in steric effects in the helix, and thus destabilizing the conformation of [*R*_p_, *R*_p_]-PT duplex, tallying with the results from the *T*_m_ values measurement above by DSC assay, and also in agreement with previously theoretical prediction^21^. Therefore, PT modification disturbs local conformation close to modification site, coincide with the results from NMR chemical shift perturbation (Fig. 2).

### The possible biological functions of PT dsDNA

Previously, the phosphorothioate DNA was suggested to act as antioxidant in diverse bacteria^14^. Our biochemical data suggests that the [*R*_p_, *R*_p_]-PT modified dsDNA has better ET potential than PT-free dsDNA, so that the *R*_p_-PT DNA is more resistant *in vivo* to the double-strand break damage caused by H_2_O_2_ than PT-free DNA, and *Salmonella enterica* cells with *R*_p_-PT DNA grow with resistance to H_2_O_2_. Furthermore, sulfur on the PT-modified DNA can be consumed and the DNA could be converted to PT-free state when the bacteria were incubated with H_2_O_2_. Thus, the *R*_p_-PT modification ensures the stability and integrity of the bacteria gene.

The activity of many restriction endonucleases is always inhibited if a PT modification is found at the cleavage site. However, in the presence of Mn^2+^ ion, ScoMcrA, the type IV restriction endonuclease, found in strain *Streptomyces coelicolor*, can specifically cleave *R*_p_-PT modified DNA by selectively targeting [*R*_p_, *R*_p_]-PT dsDNA (not [*S*_p_, *S*_p_]-PT dsDNA) and making double-strand break 16–28 nt away from the PT-modified sites^5^,^13^. Our solution structure of [*R*_p_, *R*_p_]-PT dsDNA indicates that the sulfur atoms locate in the major groove of [*R*_p_, *R*_p_]-PT dsDNA structure, which might facilitate its interaction with ScoMcrA, since the DNA-target protein generally interacts with the major groove of the dsDNA. Thus, from the structural point, the *R*_p_-PT modification is also favorable to gene stability.

Taken together, the biochemical and structural studies on PT modified DNA present the basis for how physiological [*R*_p_, *R*_p_]-PT dsDNA to correctly function in bacteria. We suggest that the biological function of PT modification is dialectical to the bacteria. On one hand, the [*R*_p_, *R*_p_]-PT DNA protects the hosting bacteria by working as antioxidant when the bacteria live with oxidants such as H_2_O_2_, on the other hand, [*R*_p_, *R*_p_]-PT DNA acts as a marker, directing restriction enzyme observed in other hosts, like ScoMcrA, to bind it and to correctly cleave the PT modified DNA, so that bacteria cannot spread and survive. This explains why PT modification is maintained in diverse bacteria.

## Methods

### DNA synthesis, purification, chirality determination of phosphorothioation

All DNA strands were commercially synthesized from Shanghai Sangon Biological Engineering Technology and Service Co. Ltd, China, with following sequences observed in the genes of *Streptomyces lividans*: 5′- C_1_G_2_^PX^G_3_C_4_C_5_G_6_C_7_C_8_G_9_A_10_-3′ with its complementary strand 5′- T_11_C_12_G_13_G_14_C_15_G_16_^PX^G_17_C_18_C_19_G_20_-3′, where X is oxygen or sulfur atom. In the whole manuscript, the dsDNA were thus named as PT-free dsDNA, [*S*_p_, *S*_p_]-PT dsDNA (*i.e*., two strands contains *S*_p_-PT modification), or [*R*_p_, *R*_p_]-PT dsDNA (*i.e.*, two strands contains *R*_p_-PT modification), respectively. The molecular weight of these strands was confirmed by running PAGE gel and MALDI TOF mass spectroscopy.

The PT-free oligomers without DMT groups at PAGE grade were dissolved in 20 ml of double-distilled water, and then purified by running semi-prep Zorbax-SAX anion exchange column (Agilent Tech Inc., USA) using buffer A and buffer B (buffer A, 40mM Na_3_PO_4_, 10% CH_3_CN, pH7.0; buffer B, 40 mM Na_3_PO_4_, 1 M NaCl, 10% CH_3_CN, pH7.0). The fractions were collected and concentrated under reduced pressure in a Speed-Vac concentrator, and subsequently desalted by running reversed phase (RP) ODS-C18 HPLC column (YMC Inc. Japan) (buffer C, also called as TEAA buffer, prepared by mixing 55 ml of N(CH_2_CH_3_)_3_ and 22 ml of acetic acid, then adding water up to 4 L, sterilized with 0.22 μM filter, buffer D, 100% CH_3_CN).

The two PT-modified oligomers were synthesized with DMT groups, the P-S bond in them are racemic, thus the strands were first processed overnight with 25% NH_4_OH overnight at 55 °C, and then concentrated with Speed-Vac concentrator, and then purified by running reverse phase (RP) ODS-C18 HPLC column, dealt with 20% (V/V) acetic acid over 16 h. The *R*_p_- and *S*_p_- PT modified strands were isolated basically by running Proteomix SAX-NP5 strong anion column (SEPAX Tech Inc, USA) (with buffer A and buffer B) (Supplemental Fig. S1), enriched so that it was enough to make NMR samples, and finally desalted by running RP ODS-C18 HPLC column (DMT-off) (with buffer C and buffer D), respectively.

To determine the chirality of phosphorus in phosphorothioate group in PT modified DNA, enzyme hydrolysis of DNA was performed as described in the previous report^2^. About 50 μg of each isolated strand was dissolved in 4 μl water, mixed with 3U snake venom phosphodiesterase (2 μl in total, *Crotalus adamanteus*, USB), 40 U alkaline phosphatase (2 μl in total, New England Biolabs), 5 μl Tris buffer (100 mM Tris-HCl, pH 8.75), 5 μl of 150 mM MgCl_2_ solution and 32.5 μl H_2_O, hydrolyzed at 37 °C. After 6 hours of incubation, the mixture was analyzed by running analytic ODS-C18 RP-HPLC with a flow rate 1.0 ml/min (buffer E, double-distilled water, buffer D, 100% CH_3_CN) following the centrifugal ultrafiltration. The *R*_p_-PT modified ssDNA was finally digested into three peaks (G, C, A or T), while the *S*_p_-PT modified ssDNA was digested into four peaks (G, C, -G^PS^G-, A or T) (Supplemental Fig. S2).

For biochemical and structural studies, the PT-free dsDNA was formed by mixing two strands without PT modification at equal molar ratio, while [*S*_p_, *S*_p_]-PT dsDNA or [*R*_p_, *R*_p_]-PT dsDNA was formed by mixing two strands with *S*_p_-PT or *R*_p_-PT modification at equal molar ratio, respectively. The mixtures were annealed by heating at 95 °C for 5 mins and slowly cooling down to room temperature.

### Thermal denaturation by differential scanning calorimetry

To determine the stability of dsDNA after PT modification, excess heat capacity (Δ*C*_P_) versus temperatures for the thermally induced transitions of the duplexes with or without [*S*_p_, *S*_p_]-PT or [*R*_p_, *R*_p_]-PT modification were measured by differential scanning calorimetry (DSC) on a Nano DSC Calorimeter (TA Instruments-Waters LLC). In the DSC experiments, the duplexes were dissolved in buffer F (10 mM sodium phosphate, pH 7.0 and 150 mM NaCl) with a final concentration of 0.9 mg/ml, the heating rate was 60 °C per hour and the maximum temperature was 95 °C. After reaching the maximum temperature, the samples were cooled at the same rate to the starting temperature of 25 °C. Each sample was scanned under pressure from 25 °C to 95 °C with 2 rescans. Here, Δ*C*_P_ is defined as excess heat capacity, which is baseline subtracted and concentration normalized. The reference scans were subtracted from the sample scans to obtain Δ*C*_P_ versus temperature profiles. Enthalpies (Δ*H*_cal_) and entropies (Δ*S*) of duplex melting were calculated from the areas under the experimental Δ*C*_P_ versus temperature (*T*) and derived Δ*C*_P_/*T* versus *T* curves, respectively, using Prism 5.0 software.

Each duplex was verified by heating-cooling-heating cycles that the melting transitions of both modified and unmodified duplexes were fully reversible^22^. The *T*_m_ values as well as enthalpy changes of duplex melting were calculated from the areas under the experimental curves, using two-state scaled model in NanoAnalyze software.

### The redox potential of DNA measured by cyclic voltammetry

To probe the antioxidation potential of PT modified DNA, we measured the redox potential of DNA by cyclic voltammetry assay (Fig. 1 and supplemental Table S1). Electrochemical characterization of the PT-free dsDNA, [*S*_p_, *S*_p_]-PT dsDNA, and [*R*_p_, *R*_p_]-PT dsDNA was achieved at a gold electrode modified by being dipped into three DNA solutions for 12 hours, respectively. The gold electrode was cleaned sequentially by polishing with an alumina (0.3 pm)/water slurry on a Mastertex polishing cloth (Beuhler) and distilled water. Cyclic voltammetry was carried out in a three-electrode cell (diameter = 60 mm, height = 90 mm) at room temperature, in which a Pt wire and a saturated Ag|AgCl electrode were used as the auxiliary electrode and the reference electrode, respectively. Experiments were performed on 0.5 mL of DNA solutions with concentration of 0.3 mM in 40 mM Na_3_PO_4_, 100 mM NaCl buffer, pH 7.0.The potential was controlled by an Autolab PGSTAT30 electrochemical galvanotation. A potential range of −0.8 to 0.8 V (vs SHE) was studied at a scanning rate of 45 mV s^−1^.

### Circular dichroism spectrum

To investigate the effects on DNA structures by PT modification, circular dichroism (CD) spectra were recorded at 25 °C on a JASCO-815 spectropolarimeter using a 1-cm path length quartz cuvette with a reaction volume of 400 μL. DNA concentration was 7 μM. The DNA oligonucleotides were prepared in a pH 7.0 buffer containing 20 mM sodium phosphate, 20 mM NaCl. The samples were heated to 95 °C for 5 min and cooled down to room temperature overnight. For each sample, an average of three scans was taken, the spectrum of the buffer was subtracted.

### NMR samples and NMR spectroscopy

For NMR experiments, all three duplexes were about 1 mM dissolved in buffer containing 40 mM sodium-phosphate buffer, 100 mM NaCl, pH 7.0, 10% D_2_O. For NMR experiments acquired in D_2_O, the samples were lyophilized twice to exchange water into D_2_O. All NMR samples were placed in 5-mm Shigemi NMR tubes.

All NMR experiments were performed on a Varian Unity Inova 600 NMR spectrometer (with cryo-probe) equipped with triple resonances and pulsed field gradients. Two-dimensional (2D) ^1^H-^1^H NOESY (with mixing times of 50 ms, 100 ms, 150 ms, 200 ms and 250 ms), TOCSY (with a mixing time of 80 ms), and DQF-COSY spectra were acquired at 25 °C on each duplex dissolved in D_2_O using a spectral width of 6100 Hz in both dimensions. The acquisition data points were set to 2048 × 512 (complex points). To assign exchangeable protons, NOESY experiments (with mixing times of 50 ms and 200 ms) were performed at 10 °C with a spectral width of 12000 Hz, and acquisition data points 4096 × 1024 (complex points). The watergate sequence was used for water suppression^23^.

All spectra were processed using the program NMRPipe^24^. The 45° or 60° shifted sine-squared functions were applied to NOESY and TOCSY spectra. The fifth-order polynomial functions were employed for the baseline corrections. The final spectral sizes are 2048 × 1024. The ^1^H chemical shifts were referenced to 2, 2-dimethylsilapentane-5-sulfonic acid (DSS). Peak assignments and integrations were achieved using Sparky (http://www.cgl.ucsf.edu/home/sparky/). The NOE peaks were integrated using the peak fitting function and volume integration of Sparky.

The ^31^P NMR spectra were collected on dsDNA samples at about 1.5 mM concentration in D_2_O (40 mM sodium-phosphate buffer, 100 mM NaCl, pH 7.0) at 25 °C at 500 MHz Varian spectrometer and were referenced to an external standard of 85% H_3_PO_4_, including the one dimensional proton-decoupled phosphorus spectrum, and two-dimensional (2D) heteronuclear ^31^P-^1^H Correlation Spectroscopy (^31^P-^1^H HETCOR). Assignments of the individual ^31^P resonance were accomplished by a combination of 2D ^1^H-^1^H NOESY, COSY, TOCSY and ^31^P-^1^H HETCOR.

### Restraints generation and NMR structural determination

The distances between the non-exchangeable protons were estimated based on the NOE cross-peak volumes at 50 ms, 100 ms, 150 ms, 200 ms and 250 ms mixing times, and were grouped into three distance ranges 1.8–2.9 Å, 1.8–3.5 Å and 1.8–6.0 Å, corresponding to strong, medium and weak NOEs, respectively. The cytosine base proton H5-H6 distance (2.45 Ǻ) was used as the reference. The distances involving the unresolved protons, e.g. methyl protons, were assigned using pseudoatom notation to make use of the pseudo-atom correction automatically computed by XPLOR-2.34 (NIH version)^25^. The exchangeable proton restraints were obtained based on NOESY data sets at two mixing times in H_2_O. Cross-peaks involving exchangeable protons were classified at strong (strong intensity at 50 ms), medium (weak intensity at 50 ms) and weak (observed only at 200 ms), and the distances between protons were then restrained into 1.8–4.0 Å, 1.8–5.0 Å and 1.8–8.0 Å, respectively.

The sugar conformation can be easily established from the information contained in the vicinal proton-proton J-coupling constants, which can be related to different dihedral angles. The values of J-coupling constants *J*_H1′-H2′_, *J*_H1′-H2″_, *J*_H2′-H3′_ and *J*_H2″-H3′_ were obtained by simulating H2′/H2″-H1′, H1′-H2′/H2″, H3′-H2′/H2″ cross-peaks using the program ACME^26^. Signal overlapping is not severe in these regions, and the cross-peaks are, except for the terminal residues, well-resolved. The first remarkable feature of these spectra was strong cross-peaks of H1′-H2′, with *J*-coupling constants close to or larger than 10 Hz, indicating a predominant S-type conformation (2′-endo) for the sugars of these duplexes.

The deoxyribose pseudorotational angles (*P*) were estimated by examining the ^3^*J*_HH_ of sugar protons^27^. The data were fit to curves relating the coupling constants to the pseudorotational angles, the sugar pucker amplitude (φ). The pseudorotation and amplitude ranges were converted to the five dihedral angles ν_0_ to ν_4_. Coupling constants measured from ^31^P-^1^H HETCOR spectra were applied to the Karplus relationship^28^ to determine the backbone dihedral angle ε (C4′-C3′-O3′-P), related to the H3′-C3′-O3′-P angle by a 120° shift. The ^3^*J*_P-H5′_ values obtained from ^1^H-^31^P HETCOR spectra were around 2–5 Hz, suggesting that the β dihedral angles of the sugar are in trans forms (*i.e*, β = 180°). The ζ (C3′-O3′-P-O5′) backbone angles were calculated from the correlation between ε and ζ in B-DNA^29^. Other backbone torsion angle restraints were using empirical data derived from B-DNA^30^. Watson-Crick hydrogen bonding constraints and plane constraints were also used to prevent twisting between base pairs.

To be different from normal guanines (GUA), during structural calculation, the *S*_p_-PT and *R*_p_-PT modified guanines were termed as two new residues SSG and RSG, respectively, whose topology files and the parameter files were home-written and integrated into XPLOR package. The arbitrary extended templates of PT-free dsDNA, [*S*_p_, *S*_p_]-PT dsDNA, and [*R*_p_, *R*_p_]-PT dsDNA were first generated based on their sequences. The structures of these three duplexes were then calculated by the program XPLOR 2.34 (NIH version)^25^. A total of ten iterations (50 structures in the initial eight iterations) were performed. 100 structures were computed in the last two iterations, 20 conformers with the lowest energy are used to represent the 3D structures. In the ensemble of the simulated annealing 20 structures, there was no distance constraint violation more than 0.4 Å and no torsion angle violation more than 5°. Their structural statistics were summarized in Table 1. All figures were generated using the program PyMOL (http://pymol.org/) and MOLMOL^31^.

## Additional Information

**How to cite this article**: Lan, W. *et al.* Structural investigation into physiological DNA phosphorothioate modification. *Sci. Rep.* **6**, 25737; doi: 10.1038/srep25737 (2016).

**Accession Code**: The coordinates of NMR solution structures of PT-free dsDNA, [Sp, Sp]-PT dsDNA and [Rp, Rp]-PT dsDNA had been deposited with RCSB Protein Data Bank under accession numbers 5J3G, 5J3I and 5J3F, respectively. The chemical shift assignments of PT-free dsDNA, [Sp, Sp]-PT dsDNA and [Rp, Rp]-PT dsDNA were also deposited with BMRB ADIT-NMR online deposition system under the accession numbers 30053, 30054 and 30052, respectively.

## Supplementary Material

Supplementary Information

## Figures and Tables

**Table 1:** Statistical analysis of NOE-based distance constraints and dihedral angle constrains used in NMR structural calculation.

	PT-free dsDNA	[*R*_p_, *R*_p_]-PT dsDNA	[*S*_p_, *S*_p_]-PT dsDNA
*Distance restraints from NOEs*			
Total NOE	495	475	434
Intra-residue	358	329	311
Inter-residue	137	149	133
Sequential (|i-j| = 1)	101	105	69
Non-sequential (|i-j| > 1)	36	44	43
Hydrogen bonds	79	79	79
*Structural statistics Violation* (*mean and SD*)			
Distance constrain (Å)	0.046 ± 0.0004	0.044 ± 0.0003	0.031 ± 0.0004
Dihedral angle constrains (°)	1.22 ± 0.01	1.18 ± 0.01	1.30 ± 0.002
*Deviations from idealized geometry*			
Bond lengths (Å)	0.002 ± 0.00002	0.002 ± 0.00003	0.002 ± 0.00004
Bond angles (°)	0.74 ± 0.0004	0.75 ± 0.0007	0.73 ± 0.0001
Impropers (°)	0.28 ± 0.002	0.26 ± 0.001	0.25 ± 0.003
*Average pairwise r.m.s.d.* (Å)			
backbone atoms	0.08 ± 0.003	0.1 ± 0.004	0.16 ± 0.005
All atoms	0.07 ± 0.003	0.09 ± 0.003	0.14 ± 0.004
