# Supplementary material for: Structural investigation into physiological DNA phosphorothioate modification
Source: Sci Rep. 2016 May 12;6:25737. doi: 10.1038/srep25737 (PMC4864419; doi:10.1038/srep25737)
Supplement: Supplementary Information [file srep25737-s1.pdf]

Structural investigations into physiological DNA phosphorothioate modification

Wenxian Lan[1], Zhongpei Hu[1], Jie Shen[1, 2], Chunxi Wang[1], Feng Jiang[3], Huili Liu[4], Dewu Long[3], Maili Liu[4], Chunyang Cao[1]*

Supplemental material and methods

PT modified DNA isolation and P-S bond stereochemistry characterization

The R_p- and S_p- PT modified single-stranded (ss) DNA were successfully isolated from their racemic mixtures by running a Proteomix SAX-NP5 strong anion-exchange column on a HPLC system. The two isolated fractions were collected and enriched so that they were enough to make NMR samples, and desalted by running ODS-C18 reverse phase column on a HPLC system. As reported in the previous literature [1], the R_p-PT ssDNA strand generally has a shorter retention time than S_p-PT DNA strand upon running SAX anion-exchange column. To characterize the stereochemistry of P-S bond in the isolated strands, the DNA hydrolysis was then performed with snake venom phosphodiesterase and alkaline phosphatase [1, 2]. The snake venom phosphodiesterase selectively digests the R_p but not S_p configuration [3]. Therefore, in our case, either the R_p-PT modified ssDNA 5'-$C_1G_2{}^{PS}G_3C_4C_5G_6C_7C_8G_9A_{10}$-3' or its complementary R_p-PT modified ssDNA 5'-$T_{11}C_{12}G_{13}G_{14}C_{15}G_{16}{}^{PS}G_{17}C_{18}C_{19}G_{20}$-3' (both with a shorter retention time than their S_p-PT strands in supplementary Figure S1) was digested into three peaks (G, C, A or T) (supplementary Figure S2), while both the S_p-PT modified ssDNA 5'-$C_1G_2{}^{PS}G_3C_4C_5G_6C_7C_8G_9A_{10}$-3' and its complementary S_p-PT modified ssDNA 5'-

$T_{11}C_{12}G_{13}G_{14}C_{15}G_{16}{}^{PS}G_{17}C_{18}C_{19}G_{20}$-3' were digested into four peaks (G, C, -G^{PS}G-, A or T) (supplemental Figure S2).

Supplemental figures and tables

Supplemental Table S1 The electron transfer potential of PT-free dsDNA, [R_p, R_p]-PT dsDNA, [S_p, S_p]-PT dsDNA and corresponding ssDNA. N.D. means 'not detectable'.

	E_{ev} (dsDNA)	E_{ev} (ssDNA)
R_p-PT	-0.62 V	-0.37 V
S_p-PT	-0.72 V	-0.43 V
PT-free	-0.28 V	N.D.

Supplemental Fig. S1 (A) The isolation of R_p-PT ssDNA (peak 1 in A, and further purification in B) and S_p-PT ssDNA (peak 2 in A, and further purification in C) by running Proteomix SAX-NP5 strong anion-exchange column two times on HPLC system, before desalting.

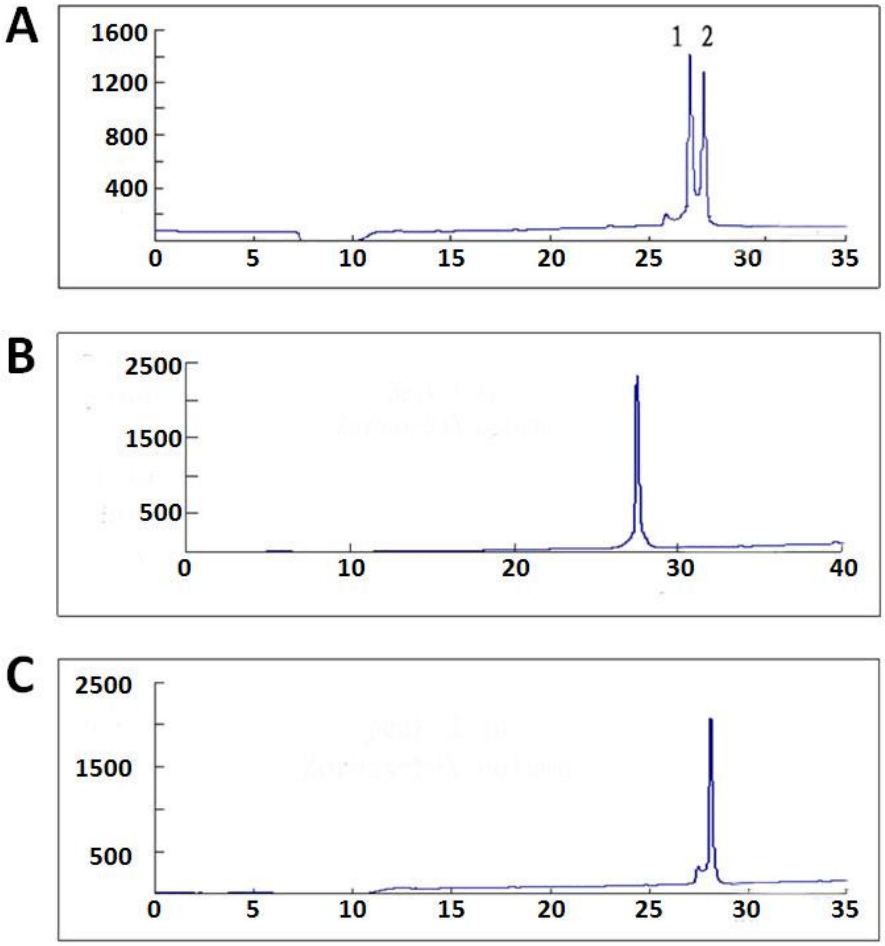

Supplemental Fig. S2 The characterization of the stereochemistry of PT bond in (A) *R*p-PT ssDNA and (B) *S*p-PT ssDNA strands firstly by DNA hydrolysis with snake venom phosphodiesterase and alkaline phosphatase, and then by running reverse phase columns on HPLC systems, respectively.

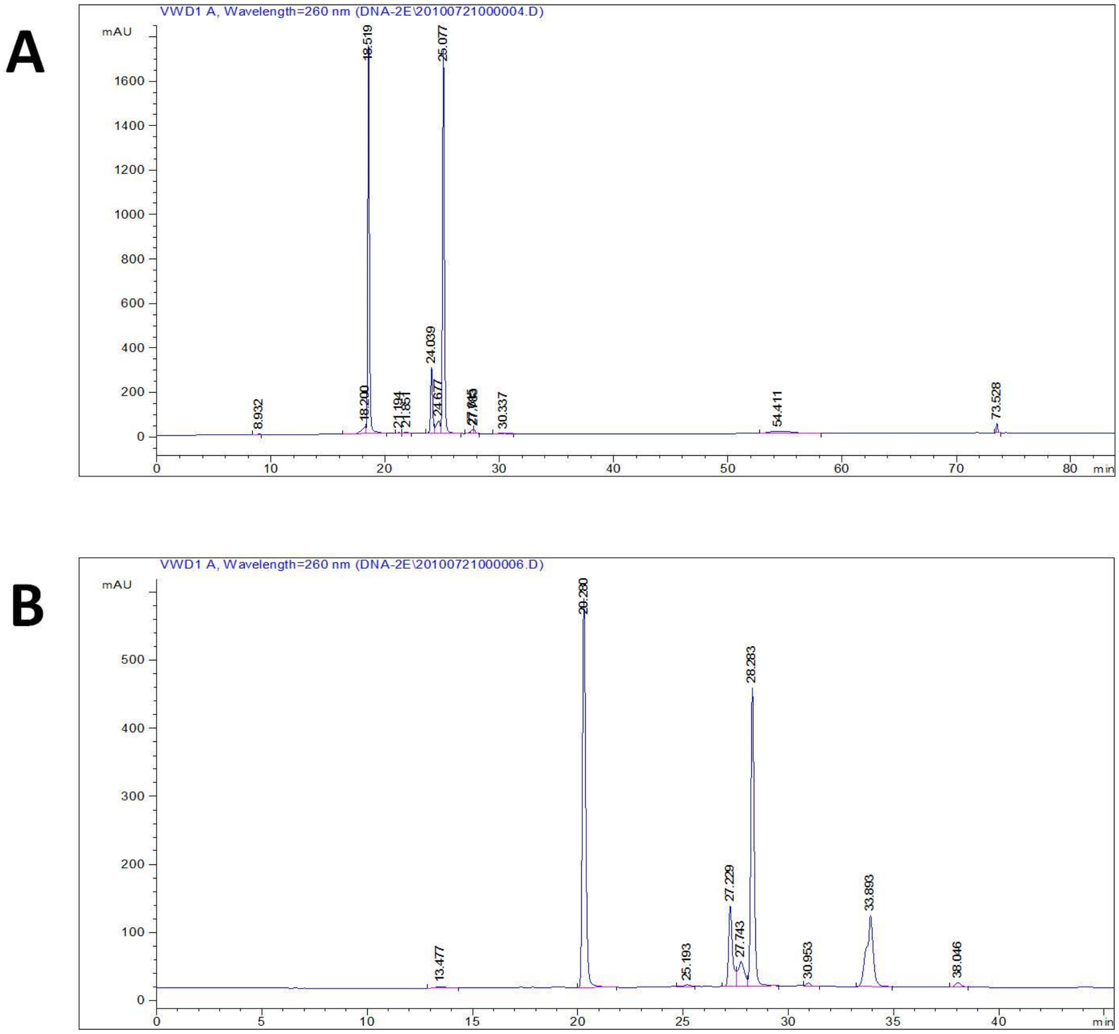

Supplemental references

1. Wang, L., et al., *Phosphorothioation of DNA in bacteria by dnd genes.* Nat Chem Biol, 2007. **3**(11): p. 709-10.
2. Pang, B., et al., *Lipid peroxidation dominates the chemistry of DNA adduct formation in a mouse model of inflammation.* Carcinogenesis, 2007. **28**(8): p. 1807-13.
3. Burgers, P.M. and F. Eckstein, *Diastereomers of 5'-O-adenosyl 3'-O-uridyl phosphorothioate: chemical synthesis and enzymatic properties.* Biochemistry, 1979. **18**(4): p. 592-6.
